# Absence of influenza vaccination among high-risk older adults in Taiwan

**DOI:** 10.1186/1471-2458-10-603

**Published:** 2010-10-13

**Authors:** Ying-Chun Li

**Affiliations:** 1 Institute of Health Care Management, National Sun Yat-Sen University, 70 Lien-Hai Road, Kaohsiung 804, Taiwan

## Abstract

**Background:**

Older adults, who often have more than one chronic disease, are at greater risk of influenza and its complications. However, because they often see physicians for other more pressing complaints, their physicians, focusing on one condition, may forget to suggest preventive measures for other diseases such as influenza. This study investigates what major factors affect an older adult with more than one chronic condition missing a vaccination opportunity.

**Methods:**

Retrospectively reviewing a nationally representative random sample of medical claims from Taiwan's National Health Insurance Research Database during the period 2004 - 2006, we first identified patients sixty-five years or older who had visited physicians. Each patient was assigned a proxy for health status, the Charlson Comorbidity Index (CCI) score. An older claimant was defined has having "absence of a vaccination" when he or she had visited a physician during an influenza season but did not receive an influenza vaccination. Multivariate logistic regression was performed to estimate how likely it would be for older adults with various CCI scores to miss a vaccination.

**Results:**

Out of 200,000 randomly selected claims, 20,923 older adults were included in our final analysis. We found older adults with higher CCIs to be more likely to have an absence of vaccination (*p *< 0.01). Our multivariate logistic regression results revealed CCI to be the greatest predictor of absence of vaccination, after controlling for individual factors and medical setting. Older adults with CCI scores three or higher were nearly five times more likely to miss a vaccination than those with a CCI of zero [OR: 4.93 (95%CI, 4.47-5.42)]. Those with CCIs of one and two were 2.53 and 3.92 times more likely to miss vaccination than those with a CCI of zero [OR 2.53 (95%CI, 2.26-2.84) and OR 3.92 (95%CI, 3.51-4.38), respectively].

**Conclusions:**

The greater the number of certain comorbid conditions, the greater the likelihood a flu vaccination will be missed. Physicians would be well advised to not let the presenting problems of older patients distract from other possible health problems that might also need attention, in this case influenza vaccinations.

## Background

Influenza is a common respiratory disease affecting large proportions of many populations annually [1,2]. There is increased likelihood of serious complications or death from influenza infection in older patients with co-existing chronic illnesses [1,3,4] and it is, therefore, recommended that these high-risk individuals receive annual flu vaccinations, which have been found to an effective preventive measure [5]. However, as was suggested by one European study, the presence of multiple chronic conditions may lead to under-treatment, possibly because a physician may focus on a presenting problem at the expense of other chronic conditions [6].

Whether or not a physician recommends vaccination strongly influences his or her patient's decision to be vaccinated [7-9]. It would be logical to assume that because older adults with multiple chronic conditions see physicians more often, they have greater opportunity for receiving vaccinations than other groups. Some older adults have been found to miss vaccination opportunities due to limited access to care in the United States [10]. However, with the implementation of its National Health Insurance program in 1995, barriers to access have been removed in Taiwan as almost all medical expenses are now covered. Influenza vaccinations have been provided free of charge to the older adults in Taiwan since 2001. The vaccination rate should be high there, but it remains low. In 2007, the average influenza vaccination rate for older adults was 49.10% [11], far below the target level of 68.0% set by Taiwan's Center for Disease Control for a 2010 national goal [12]. While there may be other reasons for this low rate, including patient resistance or lack of knowledge, this study investigates how the health status of an elderly person may affect the likelihood that he or she would miss a vaccination opportunity when seeing a physician in Taiwan.

## Methods

### Data

In this retrospective study, we first used a dataset consisting of 200,000 nationally representative randomly selected subjects from Taiwan's National Health Insurance 2004 - 2006 claims database. From these claims records, we could collect information on a claimant's age and gender, presenting problems, diagnoses, medical care facility, medical services rendered, as well as whether they received an influenza vaccination during this investigation's two study periods, the flu seasons October 2004 to April 2005 and October 2005 and April 2006. For this analysis, we selected claimants aged 65 or over with one or more of the chronic conditions considered by WHO to be in need of influenza vaccinations: diabetes, chronic lung diseases, chronic kidney disease, chronic heart disease, cancer, and immunodeficiency [2,13]. The subjects were then divided into those who received influenza vaccinations during the study period and those who missed the opportunity for the vaccination.

### Dependent Variable

A missed opportunity for vaccination was defined if a subject 65 years older had any of the above-mentioned chronic conditions and did not receive a vaccination during one of his or her visits to a physician during the study period. The subjects were assigned a dummy variable "yes" if the vaccination opportunity was missed or "no" it was not missed.

### Independent Variables

To control for provider factors, the independent variables for this study included accreditation level of health care facility (medical center, regional hospital, district hospital, or clinic), facility ownership, and whether or not they were teaching hospital. To evaluate the effect that health status might have on whether or not a claimant would receive an influenza vaccination, we further divided the subjects into four groups based on the Charlson Comorbidity Index (CCI) score [14]. The CCI is used to assign a patient a health status score based on the number and types of chronic conditions (e.g., diabetes, COPD, congestive heart failure, renal disease, liver disease, cancer) he or she has. The scores range from 0 to more than 10, based on whether a person has none or one or more of the chronic conditions included in the tool. The higher the score, the more severe the health status. Based on the distributions of all subjects according to the adapted Charlson comorbidity index scores, we analyzed the subjects with CCI scores of 0, 1, 2, and 3 or higher. We cut off CCI at 3 or more because so few patients scored higher than 3 that our distribution would be skewed.

In our statistical analysis, we first described the characteristics of the subjects and the care they received descriptively. A *p*-value < 0.05 was considered significant by using chi square tests. Multivariate logistic regression was used to estimate the likelihood that an opportunity would be missed, controlling for age, gender, CCI score, and various hospital characteristics. All statistical operations were performed using STATA 10.1 (College Station, Texas, USA).

## Results

### Missed vaccination opportunity

As can been seen in Table 1, we had a total of 20,923 observations with an average age of 73.5 year. Women made up 52.6% of the sample. There were 14,091 older adults who received influenza vaccinations during 2005-2006 flu season. There were 6,832 older adults who did not receive vaccinations during the same study period. The global vaccination coverage in the study sample was 67.35%. A significantly greater proportion of older old adults, adults with higher CCI scores, those not previously vaccinated, and those seeking medical care at medical centers, teaching hospitals, and non-for-profit hospitals, and those frequently seeking care in the internal medicine departments missed vaccination opportunities during the study period (all, *p *< 0.01). There was no gender difference.

**Table 1:** Descriptive statistics of whether elderly persons had absence of influenza vaccination at 2005-2006 flu season in Taiwan

Variables	Received Vaccination (*n *= 14091)	Had an Absence of Vaccination (*n *= 6832)	*P*- value
*Individual factors (n*, %)			
Age			
65-69	5018(68.0%)	2362(32.0%)	< 0.01
70-74	3523(68.5%)	1624(31.5%)	
75-79	2785(66.7%)	1390(33.3%)	
≥ 80	2765(65.5%)	1456(34.5%)	
Gender			
Women	7475(67.9%)	3537(32.1%)	0.08
Men	6616(66.8%)	3295(33.2%)	
Charlson Comorbidity Index			
0	3929(84.6%)	713(15.4%)	< 0.01
1	2357(71.9%)	922(28.1%)	
2	2133(62.3%)	1289(37.7%)	
≥ 3	5672(59.2%)	3908(40.8%)	
*Health care utilizations (n*, %)			
Received flu shot at 2004-2005 flu season			
No	6690(56.7%)	5117(43.3%)	< 0.01
Yes	7401(81.2%)	1715(18.8%)	
*Hospital factors (n*, %)			
Hospital level			
Medical centers	1536(57.5%)	1137(42.5%)	< 0.01
Regional hospitals	1812(59.9%)	1214(40.1%)	
District hospitals	1738(65.8%)	902(34.2%)	
Clinics	9005(71.6%)	3579(28.4%)	
Teaching status			
Teaching	3486(59.7%)	2352(40.3%)	< 0.01
Non-teaching	10605(70.3%)	4480(29.7%)	
Ownership			
Public	2324(63.3%)	1350(36.7%)	< 0.01
Non-for-profit	1616(58.4%)	1151(41.6%)	
For profit	10151(70.1%)	4331(29.9%)	
Specialty divisions			
Internal medicine	3091(59.6%)	2094(40.4%)	< 0.01
Family medicine	1499(69.7%)	614(30.3%)	
Other specialties	9501(70.9%)	4124(29.1%)	

### Regression analysis

Our multivariate logistic regression revealed that CCI score was most predictive of missed vaccination opportunity (Table 2). The greater the score, the greater the likelihood a vaccination opportunity would be missed. Those with CCI scores 3 or higher were nearly five times more likely to miss a vaccination opportunity than those with zero CCI scores [OR: 4.93 (95%CI, 4.47-5.42), *p *< 0.01]. Those with CCI scores of 1 and 2 were significantly more likely to miss a vaccination opportunity than those with CCI scores of 0 [OR 2.53 (95%CI, 2.26-2.84), *p *< 0.01 and OR 3.92 (95%CI, 3.51-4.38), *p *< 0.01, respectively], after controlling for individual and medical setting factors. In addition, older adults who did not receive influenza vaccination during the 2004-2005 flu season were significantly more likely to miss a vaccination during the following flu season [OR 4.26 (95%CI, 3.97-4.56), *p *< 0.01].

**Table 2:** Adjusted odds ratio of the probability of absence of influenza vaccination among elderly persons with multiple chronic diseases

Variables	Odds Ratio	95% CI	*P*-value
*Individual factors*			
Age			
65-69	1		
70-74	1.14	[1.05 -1.24]	< 0.01
75-79	1.14	[1.04 -1.25]	< 0.01
≥ 80	1.07	[0.98-1.17]	0.19
Gender			
Men	1		
Women	1.14	[1.07 -1.22]	< 0.01
Charlson Comorbidity Index			
0	1		
1	2.53	[2.26 - 2.84]	< 0.01
2	3.92	[3.51 - 4.38]	< 0.01
≥ 3	4.93	[4.47 - 5.42]	< 0.01
*Health care utilizations*			
Received flu shot at 2004-2005 flu season			
Yes	1		
No	4.26	[3.97 - 4.56]	< 0.01
*Hospital factors*			
Hospital level			
Medical Center	1		
Regional hospitals	0.93	[0.83 - 1.05]	0.43
District hospitals	0.78	[0.67 - 0.91]	< 0.01
Clinics	0.84	[0.72 - 0.98]	< 0.01
Teaching status			
Teaching	1		
Non-Teaching	0.98	[0.87 - 1.11]	0.27
Ownership			
Public	1		
Non-for-profit	1.03	[0.92 - 1.15]	0.56
For profit	0.91	[0.83 - 1.01]	0.61
Specialty divisions			
Internal medicine	1		
Family medicine	0.82	[0.73 - 0.93]	< 0.01
Other specialties	0.80	[0.75 - 0.87]	< 0.01

Other variables were also found by multivariate logistic regression to significantly affect the likelihood that a vaccination would be missed: increased age and being female had a positive effect and facility accreditation and physician specialty had a negative effect. However, the ORs for these variables ranged between 0.78 and 1.14, meaning that they had less of an influence on the likelihood that a vaccination opportunity would be missed than CCI.

## Discussion

This study found the greater number of chronic diseases older adults had, the greater the likelihood that they would miss an influenza vaccination. We did, however, find an increased likelihood that one would receive a vaccination if that individual had had such a vaccination in the previous season.

That older people with chronic diseases are more likely to miss vaccinations suggests that even under Taiwan's National Health Insurance program, which makes it possible for these individuals to visit health care professionals as many times as they need for the care of their chronic conditions, older people may not receive the preventive care they need. Several factors might be related to this reduced likelihood. First, patients in Taiwan are more likely to visit physicians to treat diseases, not prevent them. As a result, physicians also tend to focus more attention on treating their presenting problems than preventing disease. Second, physician workload may be a factor. One Canadian study reported that physicians with heavy workloads tend to overlook the need to address disease prevention [15]. In the United States, too, physicians have been found to overlook recommending influenza vaccination to their elderly and high-risk patients [4]. In Taiwan's health care settings, the patient waiting room time is long and physicians are pressured to speed up the visits. Physicians with patients with a greater number of chronic conditions spend a longer time discussing and treating each condition, leaving little time for them to introduce a new concern and a need for prevention into the visit, though a lack of knowledge regarding the important and usefulness may also be a factor [16,17]. Finally, some physicians may fear adding further burden to the weakened immune systems of older patients with multiple chronic diseases. These reasons are conjecture and may, therefore, need further substantiation.

The study also found that older adults who received influenza vaccinations at year 2004-2005 flu season were significantly more likely to receive vaccinations the following year, which is consistent with previous studies [18,19]. Based on these findings, it might be assumed that campaigns encouraging older adults to receive influenza vaccination may have a snowball effect, bringing about cumulative increase in vaccination rates year by year.

This study found that older persons were more likely to obtain vaccinations at district hospitals and clinics than at medical centers, a finding similar to a study reported that missed opportunities were more likely to occur in facilities providing integrated services [20]. Patients seeking care at larger centers with integrated care facilities may have more severe conditions requiring more immediate attention, and thus there may be a greater chance that the prevention for less immediate problems would be overlooked. We also found that family medicine departments were more likely to prescribe vaccinations than internal medicine departments. The designation "family practitioner" used in Taiwan can generally be equated with general practitioners in other countries, and internal medicine departments in Taiwan usually consist of many different internal medicine specialties. Internists have also been found elsewhere to be lacking in adequate attention to preventive medicine [15]. Since we were unable to link specific physician with certain older adult's influenza vaccination, we analyzed the physician specialty as the proxy of physician influence on vaccination.

Some specific diseases listed in the CCI were associated to greater missed opportunity of influenza vaccination. For example, a greater proportion of older adults with heart failure and chronic obstructive pulmonary disease (COPD) did not receive influenza vaccinations during the study period. Further research on vaccination for these chronic diseases among older adults is needed.

This study is limited in that it lacks detailed information regarding encounters between physicians and patients. Claims data only cover what was diagnosed and what was prescribed. Therefore, if no vaccination was performed, then it is difficult to know with certainty whether possible vaccination was discussed.

## Conclusions

In summary, older persons with more chronic diseases are more likely to miss opportunities to receive influenza vaccination. Receiving a vaccination one season increased the likelihood that a person would receive a vaccination the following season. We conclude that free vaccine coverage does not always greatly increase vaccination rates, particularly for those older adults with multiple chronic conditions. Therefore, further research is needed to find ways to increase the vaccination rate in this population. Considering that physicians mostly concerned with treatment of presenting symptoms may forget to recommend flu vaccinations, policymakers may want to consider establishing a system that allows physicians to see a patient's preventive care history when their records are retrieved during their visits. Physicians may also need to be reminded of the importance of preventive measures for this population. Efforts such as these may help increase the vaccination rates and better protect the health of this vulnerable population.

## Competing interests

The author declares that they have no competing interests.

## Authors' contributions

YCL designed the project, contributed to the data acquisition, data analysis and interpretation, and wrote the manuscript.

## Pre-publication history

The pre-publication history for this paper can be accessed here:

http://www.biomedcentral.com/1471-2458/10/603/prepub
